# Beyond the Clinic: The Importance of Department of Defense Respiratory Viral Panel Testing for Public Health Surveillance and Force Health Protection

**Published:** 2025-04-20

**Authors:** Aileen C. Mooney, Simon D. Pollett, Brian K. Agan, Dara A. Russell, Marissa K. Hetrich, David R. Tribble, Timothy H. Burgess, Robert J. O'Connell, Rhonda E. Colombo, Kathleen E. Creppage, M. Shayne Gallaway

**Affiliations:** Global Emerging Infections Surveillance Branch, Armed Forces Health Surveillance Division, Public Health Directorate, Defense Health Agency, Silver Spring, MD: Ms. Mooney, Ms. Russell, Ms. Hetrich, Dr. Creppage, CDR Gallaway; Infectious Disease Clinical Research Program, Department of Preventive Medicine and Biostatistics, Uniformed Services University of the Health Sciences, Rockville, MD: Dr. Pollett, Dr. Agan, Dr. Tribble, Dr. Burgess, COL O'Connell, Dr. Colombo; Henry M. Jackson Foundation for the Advancement of Military Medicine, Inc., Bethesda, MD: Dr. Pollett, Dr. Agan, Dr. Colombo; Department of Medicine, Uniformed Services University of the Health Sciences, Bethesda: Dr. Colombo

Historically, military populations have been at high risk for acute respiratory infections, primarily among recruits and deployed personnel due to frequent exposures to crowded conditions, deployments, and stressful work environments. ^1 , 2^ Respiratory pathogen surveillance is critical for force health protection and clinical decision-making.

The Global Emerging Infections Surveillance Branch (GEIS) of the Armed Forces Health Surveillance Division respiratory infections focus area supports routine molecular and genomic public health surveillance of respiratory pathogens in military and non-military settings where U.S. service members may come into contact with host nation civilians. Rapid detection of specific etiologic agents within a subset of clinical samples, residual samples, or in support of an outbreak ^3 , 4^ can directly enable action to reduce transmission and maintain readiness of military members, including decisions about preventive measures, medical countermeasures, and resource allocation to safeguard the health and readiness of U.S. service members, their families, and allied forces.

Early disease diagnosis can reduce likelihood of increased disease severity and prolonged recovery. Illnesses caused by respiratory viruses can affect anyone, but illness severity may be greater for older adults, young children, individuals with compromised immune systems, people with disabilities, and those who are pregnant. ^5^ Seasonal respiratory viral infections, such as influenza and respiratory syncytial virus (RSV) exhibit distinct patterns that can be anticipated. ^6^ In regions with temperate climates, seasonal epidemics occur mainly during winter, while tropical regions tend to experience more sporadic epidemics throughout the year. ^7^

This editorial evaluates the clinical utility of increasingly common respiratory viral panel (RVP) diagnostic assays and discusses how these RVPs can improve support for force health protection and Military Health System (MHS) beneficiary public health surveillance.

## Clinical Utility of Respiratory Viral Panels

Clinical RVPs typically use a single patient sample to run tests for common viral and bacterial infections. A RVP may refer to commercial multiplex systems or laboratory tests developed in-house. Commercial RVP multiplex systems typically include a testing platform and associated consumables, making them attractive options for high volume diagnostic laboratories. ^8^

RVP molecular assays yield rapid results with high sensitivity and specificity for the most common circulating respiratory pathogens, rendering them invaluable in conjunction with clinical evaluation. Results can be obtained within a few hours depending on the specific panel and pathogens tested. While there are instances (e.g., a known outbreak or period of elevated incidence) where a rapid diagnostic test or singleplex assay may be preferred, using a RVP (i.e., multiplex test) can potentially reduce delays in result reporting compared to sequential singleplex approaches. Use of an RVP may not always be the most cost-effective diagnostic within every clinical setting.

Infections caused by non-influenza respiratory viruses (e.g., SARS-CoV-2, rhinovirus) can mimic influenza illness symptomology, particularly during periods of high influenza activity, making clinical differentiation challenging. ^9^ A health care provider may infer the cause of a respiratory infection based on the season, presentation and medical history, and in some cases, recent travel, but typically cannot conclusively differentiate between most respiratory viruses without further diagnostic testing. ^10^ Further, co-circulation and co-infection of multiple respiratory viral pathogens can contribute to uncertainty regarding the etiology of respiratory infections.

Using RVP multiplex testing in a clinical setting helps ameliorate diagnosis and treatment challenges and may enhance patient care. Identifying the specific respiratory viral pathogen enables early antiviral treatment in influenza and SARS-CoV-2 cases. Early use of influenza antivirals, such as oseltamivir or baloxavir, may reduce symptom severity and risk of complications in addition to limiting transmission. ^11^ Antivirals such as remdesivir and nirmatrelvir/ritonavir have been shown to reduce clinical severity in certain subsets of COVID-19 patients if administered early in the disease course. ^12^ RVP testing can also inform management decisions for limiting infection transmission, including antiviral chemoprophylaxis to reduce secondary attack rates in influenza cases, especially among unvaccinated individuals in congregate settings.

## Respiratory Viral Panels for Public Health Surveillance and Force Health Protection

**FIGURE 1.**
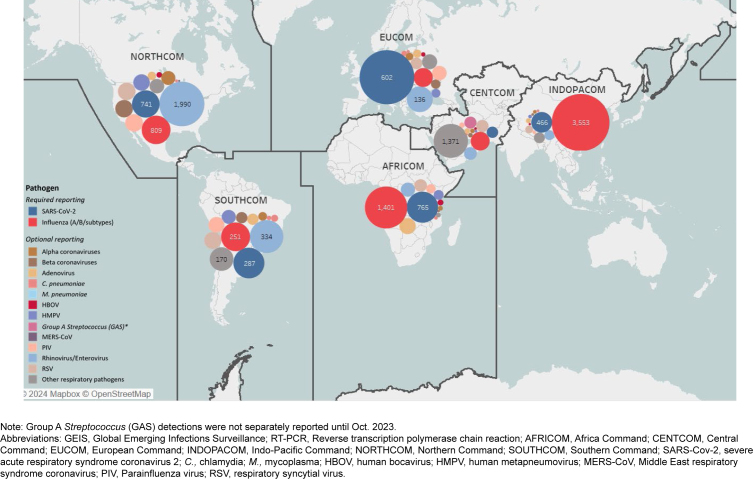
Total Number of Respiratory Pathogens Detected by GEIS-funded Laboratories Using RT-PCR, Specimen Collection Dates June 1, 2023—May 31, 2024

GEIS supports a global network of highly qualified DOD service laboratories in key locations, both domestically and internationally, to provide direct infectious disease surveillance and outbreak response. The majority of GEIS partner laboratories (GEIS-PLs) perform respiratory pathogen diagnostic testing using a RVP (or RVP in combination with singleplex testing) among U.S. service member, civilian, and foreign military and foreign national populations meeting a specific case definition for severe acute respiratory infection (SARI) or influenza-like illness (ILI). ^13^ To ensure ongoing surveillance results can be incorporated and used in a timely fashion, the GEIS respiratory infections focus area requires GEIS-PLs to report recent molecular testing detection data monthly for all pathogens included on the RVP (e.g., influenza, SARS-CoV-2, novel coronaviruses, RSV, adenoviruses, rhinoviruses, etc.). Monthly results are reported by 10 GEIS-PLs within all global combatant commands (GCCs) **(Figure 1)** .

Surveillance case definitions for ILI and SARI vary slightly among GEIS-PL protocols but are generally characterized by the presence of a fever and a cough or sore throat in the absence of a known cause other than influenza; the SARI case definition typically also requires hospitalization. Case definitions for ILI and SARI surveillance are not necessarily intended to capture all cases but describe trends over time. A variety of pathogens can cause SARI and ILI, and these are monitored closely to identify seasonal trends and describe the temporal and geographic circulation patterns (including trend deviations and outbreaks). This close monitoring is important because SARIs and ILIs are particularly problematic in some military environments (e.g., recruit training, shipboard populations, deployment settings).

GEIS-PLs routinely test samples from symptomatic persons meeting syndromic case definitions for ILI or SARI to identify circulating viruses and facilitate detection of new strains through laboratory testing and characterization as well as sharing samples with GEIS laboratories. Aggregation of standard RVP reporting (i.e., GEIS-PL monthly reports) and routine distribution of most current genomic sequencing results (from the Department of Defense Global Respiratory Pathogen Surveillance Program and Naval Health Research Center) helps continuously inform senior leaders, force health protection officers, and medical personnel of the most relevant respiratory infections circulating and their decisions for treatment and quarantine. Surveillance findings indicating serious or immediate threats necessitating a change to force health protection posture or indicating that a unit is non-mission capable due to acute health issues are reported to GEIS immediately and disseminated to relevant GCC points of contact within 24 hours. Related, surveillance findings from outbreak events may result in local policy changes related to medical countermeasures ^4^ or preventive measures. ^3^

Between June 1, 2023 and May 31, 2024, GEIS-PLs reported results from RVP (and singleplex) sample testing that detected 42,430 SARS-CoV-2 (10% positivity), 43,606 influenza (16% positivity), and 23,704 other (31% positivity) respiratory pathogens **(Table)** . The most common other respiratory pathogen detected was rhinovirus/enterovirus (17% positivity). During that period, the highest number of samples tested were submitted by U.S. Africa Command (AFRICOM), and highest percent positivity for influenza was reported by U.S. Indo-Pacific Command (INDOPA-COM), for SARS-CoV-2 by Europe Command (EUCOM), and Southern Command (SOUTHCOM) for other respiratory pathogens.

**TABLE.** RT-PCR Results from GEIS-funded Laboratories by Global Combatant Command, Specimen Collection Dates June 1, 2023–May 31, 2024

U.S. Combatant Command	Influenza			SARS-CoV-2			Other Respiratory Pathogens		
	Samples Tested (n)	% Positive	Positivity per 100,000	Samples Tested (n)	% Positive	Positivity per 100,000	Samples Tested (n)	% Positive	Positivity per 100,000
AFRICOM	14,639	9.6	9,570	19,019	4	4,022	3,305	21.9	21,876
CENTCOM	5,667	7.4	7,358	5,653	2.7	2,707	5,415	38.1	38,098
EUCOM	2,378	3.1	3,112	2,919	20.6	20,624	2,373	13.7	13,696
INDOPACOM	9,245	38.4	38,432	6,426	7.3	7,252	1,534	26.3	26,336
NORTHCOM	9,341	8.7	8,661	6,086	12.2	12,175	9,339	39.6	39,640
SOUTHCOM	2,336	10.7	10,745	2,327	12.3	12,333	1,738	48.9	48,849

Abbreviations: RT-PCR, Reverse transcription polymerase chain reaction; GEIS, Global Emerging Infections Surveillance; AFRICOM, Africa Command; CENTCOM, Central Command; EUCOM, European Command; INDOPACOM, Indo-Pacific Command; NORTHCOM, Northern Command; SOUTHCOM, Southern Command.

## Molecular Influenza Surveillance to Inform Wider Public Health Surveillance Efforts

Influenza viruses detected through public health surveillance using a RVP (or singleplex) are further analyzed by GEIS-PLs to inform selection of the specific strains for the Northern Hemisphere influenza vaccine (mandatory for service members) for the following season. A combination of epidemiologic analyses, genetic sequencing, and advanced characterization is used to generate a detailed summary of the annual influenza global landscape observed through DOD respiratory surveillance. Geographic distribution of the influenza sequences characterized, and influenza subtype ratios, are examined for the U.S. and each country surveilled.

**FIGURE 2.**
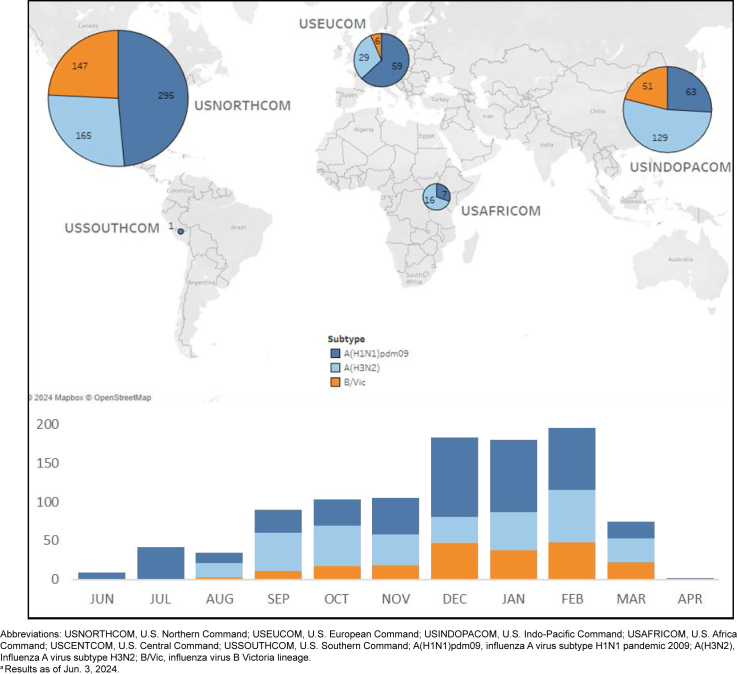
Influenza Subtype Temporal Trends ^a^ and Distribution Among Global Combatant Commands, Specimen Collection Dates June 1, 2023–April 31, 2024

For the 2023-2024 respiratory season, GEIS comprehensive analyses included positive samples and molecular sequencing data submitted to the Defense Global Respiratory Pathogen Surveillance Program (DoDGRPSP) from 10 GEIS-PLs and more than 100 DoDGRPSP sentinel sites. These findings were presented during the influenza Vaccines and Related Biological Products Advisory Committee (VRBPAC) meeting. ^14^ **Figure 2** shows the influenza sub-type geographic distribution and temporal trends from June 2023 through April 2024.

Influenza subtype ratios for the U.S. **(Figure 2)** showed a higher proportion of influenza A(H1N1)pdm09 in the northern, western, and eastern regions of the country, higher influenza A(H3N2) in the central U.S., and a higher amount of influenza B/Victoria in the southern U.S. compared to northern regions. A notably higher proportion of influenza A(H3N2) was observed in AFRICOM and INDOPACOM, while influenza A(H1N1)pdm09 was higher in EUCOM. A smaller proportion of influenza B/Victoria was observed in most regions outside the U.S. Regional trends should be interpreted with consideration of potential limitations associated with sampling or ascertainment bias (i.e., collected samples may not fully represent all persons in these populations and must be considered in context with other surveillance data collected by interagency partners). ^15 , 16^

Closely monitoring influenza infections can inform vaccine decision-making with broad global implications. During the 2023-2024 respiratory season, a combination of 3 influenza subtypes (A[H1N1]pdm09, A[H3N2], B/Victoria) were observed, with no confirmed detections of circulating influenza B/Yamagata since March 2020. Based on those (and similar) data, there was agreement during the March 2024 VRBPAC meeting to transition from a quadrivalent vaccine (which included the Yamagata strain) to a trivalent vaccine only for U.S. use, starting in the 2024-2025 respiratory virus season. ^17^ Quadrivalent influenza vaccines for distribution outside the U.S. still included the B/Yamagata as the second influenza B strain for the 2024-2025 season. ^18^

## Limitations and Future Directions

While the GEIS network is critical for continuously monitoring respiratory infections that affect service members globally, this global network of laboratories contends with several limitations. Each GEIS-funded laboratory has different priorities and surveillance populations that determine their surveillance activities, which may result in differential applications of molecular testing for respiratory pathogen detection. While the majority of GEIS-PLs currently use a RVP (or a RVP in combination with singleplex testing), they are not formally required to test for all respiratory pathogens besides influenza and SARS-CoV-2. Likewise, there are no requirements specifying which RVP (when in use) must be used for surveillance purposes among GEIS-PLs. GEIS-PLs may acquire and implement a new RVP during the lifecycle of a project, which can affect the numbers and types of pathogens that may be detected and reported to the GEIS program office, since not all RVPs are standardized.

Because these results reflect surveillance data reported directly to the GEIS program office by funded GEIS-PLs, it is possible these data under-represent the true incidence for the respiratory pathogens reported. Similarly, influenza sequencing analyses are based on samples and data submitted by sentinel sites or shared by GEIS-PLs with the DoDGRPSP. Only a small proportion of all respiratory infection samples were submitted by sentinel sites, and not all GEIS-PLs were able to contribute influenza samples or sequencing data. While GEIS RVP (and singleplex) data provide a unique global surveillance perspective of laboratory partners actively conducting respiratory surveillance, they might not accurately reflect a complete representation for all DOD active component personnel globally or within MHS.

Several low- and middle-income countries lack the resources or capabilities for widespread RVP testing, limiting respiratory surveillance that would otherwise inform diagnostics and treatment selection and preventive measures for MHS beneficiaries deployed to these areas. The GEIS network helps fulfill this need with respiratory surveillance through its network of partner laboratories in countries such as Tanzania and Djibouti, where RVP testing may otherwise be scarcely used or reported. ^19^ Deployment of RVP testing may be challenging in limited resource or forward operating areas, although possible in some early role care levels. In many austere settings, there remains a need for focused local or regional RVP surveillance to improve pre-test probability estimations.

Studies both within and outside the MHS suggest that RVPs may not always identify pathogen etiology. ^20 - 22^ Consequently, some GEIS-PLs identify a subset of SARI cases that have tested negative on a RVP for all pathogens and characterize those samples further using clinical metagenomics sequencing for public health surveillance. Metagenomic sequencing is the process of sequencing all genetic material in a sample (often using agnostic or semiagnostic sequencing) to determine the possible infecting organism without *a priori* knowledge of a specific pathogen. ^23^ Clinical metagenomics has diagnostic applications for lower respiratory tract infections and has shown promise, although it is not yet widely available, cost-efficient, nor suitable to inform routine clinical care for ILI / SARI cases. Metagenomics has substantial resource requirements including wet laboratory and bioinformatic resources. ^24^ In addition, one challenge of metagenomics in clinical practice is differentiation between clinically relevant pathogens and incidental respiratory tract colonizers, including the transient virome. ^25^ GEIS-funded respiratory surveillance activities continue to delineate which residual, pathogen-negative clinical specimens may benefit from secondary agnostic or semi-agnostic metagenomic sequencing, assessing semi-agnostic platforms that may extend diagnostic yield beyond RVP results while minimizing metagenomic ‘noise’.

Key GEIS-PLs share in-depth findings dynamically displayed on their respective dashboards, accessible to DOD partners via Carepoint, including the DoD Global Respiratory Pathogen Surveillance Program (>100 DOD sentinel sites) and the Naval Health Research Center Febrile Respiratory Illness (US-Mexico border, recruit sites). Notably, the Defense Center for Public Health-Portsmouth (DCPH-D) recently created the DHA Influenza Dashboard that includes influenza surveillance findings across the MHS.
